# Circumferential laryngopharyngectomy with free jejunal transfer for hypopharyngeal cancer: a retrospective cohort analysis of outcomes and complications

**DOI:** 10.3389/fsurg.2026.1822225

**Published:** 2026-06-24

**Authors:** Alissa De Baets, Davide Di Santo, Ann Goeleven, Katarina Segers, Thomas Nevens, Albert Wolthuis, Vincent Vander Poorten, Jeroen Meulemans

**Affiliations:** 1 Otorhinolaryngology, Antwerp University Hospitals (UZA), Edegem, Belgium; 2 Otorhinolaryngology, Head and Neck Surgery, University Hospitals Leuven, Leuven, Belgium; 3 Department of Oncology, Section Head and Neck Oncology, KU Leuven, Leuven, Belgium; 4 Otorhinolaryngology, Head and Neck Surgery, Swallowing Clinic, University Hospitals Leuven, Leuven, Belgium; 5 Plastic and Reconstructive Surgery, University Hospitals Leuven, Leuven, Belgium; 6 Department of Abdominal Surgery, University Hospitals Leuven, Leuven, Belgium

**Keywords:** circumferential defect, complications, free jejunal transfer, hypopharyngeal squamous cell carcinoma (HSCC), laryngopharyngectomy, outcomes

## Abstract

**Purpose:**

Hypopharyngeal squamous cell carcinoma (HSCC) is often diagnosed at an advanced stage and characterized by poor prognosis. While primary (chemo)radiotherapy is frequently preferred for early and intermediate stage disease, total laryngopharyngectomy (TLP) remains the gold standard for the primary treatment of locally advanced tumors and for salvage treatment of residual and recurrent HSCC. However, reconstruction of a circumferential hypopharyngeal defect is challenging. This retrospective cohort analysis evaluates the oncological and functional outcomes of TLP with free jejunal transfer (FJT).

**Methods:**

22 patients with histopathologically confirmed HSCC requiring TLP with FJT were included. Data on patient demographics, tumor characteristics, and functional and oncological outcomes were analyzed with overall survival (OS) as primary outcome.

**Results:**

Overall, 22 patients were included (18.2% female, mean age 65.6 years). Mean follow-up time was 3.9 years. Primary and salvage TLP was performed in 8 (36.4%) and 14 (63.6%) patients respectively. All primary tumors were stage IV. In the salvage group, patients had disease stage II (*n* = 1, 7.1%), III (*n* = 5, 35.7%) and IV (*n* = 8, 57.1%). Successful FJT was achieved in 95.5% of patients with only 1 FJT failure (4.5%). Pharyngocutaneous fistula (PCF) formation was the most common complication and was observed in 6 patients (27.3%); 2 were managed conservatively and 4 required surgical intervention. No in-hospital deaths were encountered. Two- and five-year OS rates were 52.3% and 26.9%, respectively. Disease-specific survival (DSS) rates were 54.7% and 42.8% respectively. Complete oral intake and voice rehabilitation were achieved in 68.2% and 52.3% of patients respectively.

**Conclusion:**

FJT deserves its place in the reconstructive armamentarium following TLP. Oncological outcomes are good and functional outcomes are acceptable but there is room for improvement.

## Introduction

Squamous cell carcinoma of the hypopharynx (HSCC) is an aggressive malignancy that represents 3%–5% of all head and neck cancers. Due to the often advanced stage at diagnosis and its tendency for distant metastatic spread, HSCC is characterized by a poor prognosis, with 5-year survival rates of less than 50% (1–3). Over the decades, there has been a shift in the primary treatment of HSCC from radical surgery to organ-preserving approaches such as radiotherapy (RT) and chemoradiotherapy (CRT). However, in advanced cases where both laryngeal and hypopharyngeal functions are already compromised, the choice of (chemo)radiation may preserve the organ but function preservation remains doubtful. In patients with extensive disease (stage IVa/b), radical surgery with appropriate reconstruction of the digestive tract and adjuvant therapy is considered the gold standard. In addition, radical salvage surgery is often the only remaining treatment in case of residual tumor or local/locoregional recurrence after initial RT/CRT (1–6). Surgery often entails a total laryngopharyngectomy (TLP), resulting in a circumferential hypopharyngeal defect that requires tubular reconstruction connecting the upper part of the oropharynx to the esophagus to restore the upper digestive tract. Multiple reconstructive options exist, including free jejunal transfer (FJT), colon transposition, gastric pull-up/gastric transposition, gastro-omental flap, and tubulated (fascio) cutaneous free flaps (FCFF). (5) The abundance of reconstructive options, none of which is ideal, leads to a complex decision-making process, aimed at selecting the optimal reconstructive strategy for the individual patient. In many centers, gastric transposition is the preferred method for reconstruction after total laryngopharyngo-esophagectomy for advanced hypopharyngeal tumors with extension into the cervical esophagus below the upper esophageal sphincter (UES). However, for TLP defects with the caudal margin located superior to the lower border of the UES, FCFF and FJT are considered the best reconstructive options (5, 7). The purpose of this retrospective case series was to evaluate the oncological and functional outcomes after TLP with FJT reconstruction for primary and salvage HSCC.

## Patients and methods

### Study design

This study was conducted as a monocentric retrospective cohort analysis of patients treated with primary or salvage TLP and FJT reconstruction for HSCC at a tertiary care academic institution (University Hospitals Leuven, Belgium), between 2000 and 2023. The study was approved by The Research Ethics Committee UZ/KU Leuven (study number MP029752) and was caried out in accordance with the ethical standards set forth in the 1964 Declaration of Helsinki and its later amendments. The collection, processing, and disclosure of personal data were performed in compliance with the General Data Protection Regulation (GDPR). For this project, data were systematically extracted from electronic patient health records and stored in the Research Electronic Data Capture (REDCap, Vanderbilt University, Nashville, USA) system. The database was structured to include a wide range of variables, including patient, tumor and treatment characteristics, complications, and oncological and functional outcomes. Tumors were retrospectively restaged according to the 8th edition of the Union for International Cancer Control (UICC) TNM classification (8).

### Patients

Strict inclusion and exclusion criteria were used to retrospectively select patients. Patients with a histopathologically confirmed diagnosis of HSCC who underwent TLP with FJT reconstruction between 2000 and 2023 were included. Patients who underwent primary surgical treatment as well as those who received salvage surgery following prior (chemo)radiotherapy were included. Complete medical records were required to be available, including preoperative imaging, surgical details, and oncological/functional follow-up. Patients were excluded if FJT reconstruction was performed for:

1. non-oncological indications (e.g., reconstruction of a neopharyngeal stenosis after TLP)
2. salvage reconstruction after previously failed reconstruction of a TLP defect (e.g., necrosis of the pharyngogastric anastomosis after gastric pull-up reconstruction)
3. reconstruction after TLP for non-SCC hypopharyngeal malignancies.

Patients meeting the inclusion criteria were, in order to reduce selection bias, consecutively included except of 2 patients with insufficient follow-up data.

Prior to surgery, all patients underwent a complete oncological work-up to assess the extent of disease and to determine the feasibility of surgery. Magnetic resonance imaging (MRI) or contrast-enhanced computed tomography (CT) of the neck was performed to assess lymph node involvement and local tumor invasion. In addition, CT of the chest and abdomen or whole-body positron emission tomography (PET-CT) was performed to exclude distant metastases. Direct laryngopharyngoscopy with biopsies was part of the standard work-up, both for achieving a histopathological diagnosis and for evaluation of the extent of the tumor in the hypopharynx and esophagus, often determining the choice of the reconstructive modality. When the hypopharyngeal tumor extends below the inferior border of the upper esophageal sphincter into the esophagus, laryngopharyngo-esophagectomy with gastric transposition is generally considered the preferred reconstruction modality. However, FJT reconstruction is technically feasible in cases of minimal esophageal extension above the level of the thoracic inlet, as this still allows achievement of adequate resection margins and jejunoesophageal anastomosis. Moreover, a flexible esophagogastroscopy was routinely performed to exclude second primary esophageal tumors. The final treatment plan was determined through multidisciplinary tumor board discussion for each patient.

Among patients eligible for inclusion, two patient groups were defined according to surgical indication: the first group consisted of patients who underwent primary TLP + FJT for previously untreated HSCC. In this primary treatment group, TLP with FJT was mainly performed in patients with locally advanced disease, typically those with cT4a HSCC with tumor invasion of the thyroid and/or cricoid cartilages. It was also considered for patients with cT3 HSCC, particularly when the tumor caused fixation of the hemilarynx or extended into the pre-epiglottic space. In contrast, cT4b tumors, characterized by invasion of the prevertebral fascia, mediastinal involvement, or encasement of the carotid artery, were generally considered unresectable. The second group included patients who underwent salvage TLP + FJT for residual or recurrent disease following prior (chemo)radiotherapy, or for patients with a second primary HSCC after previous irradiation for another head and neck malignancy. Residual disease is defined as tumor in the same anatomic (sub)site as the primary tumor, diagnosed without or within a disease free interval (DFI) of less than 6 months.

### Surgical procedure

**Figure 1:**
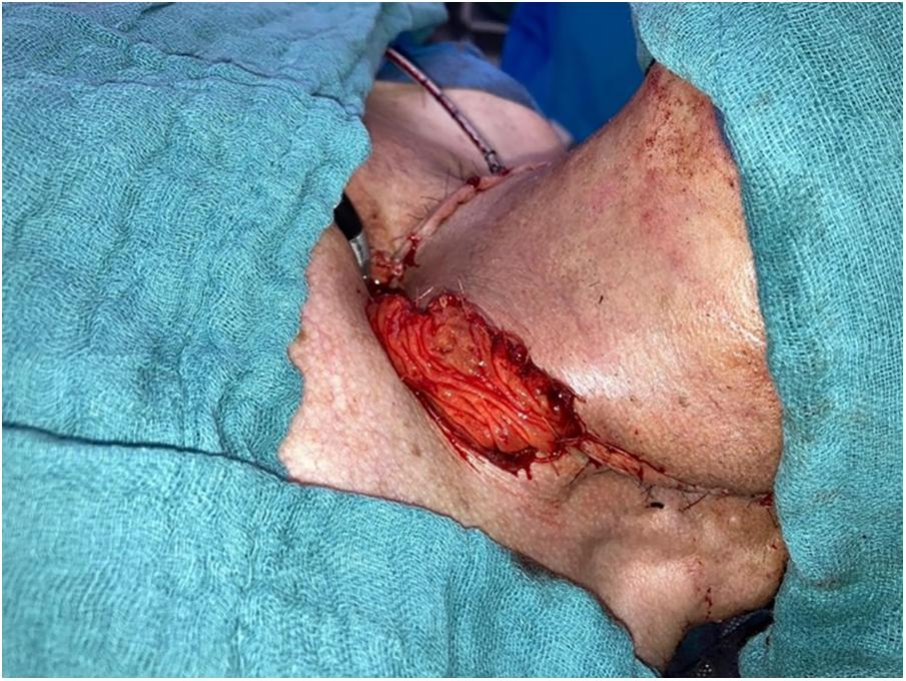
External jejunal flap for postoperative monitoring of jejunal flap viability, sutured laterally in the neck.

**Figure 2:**
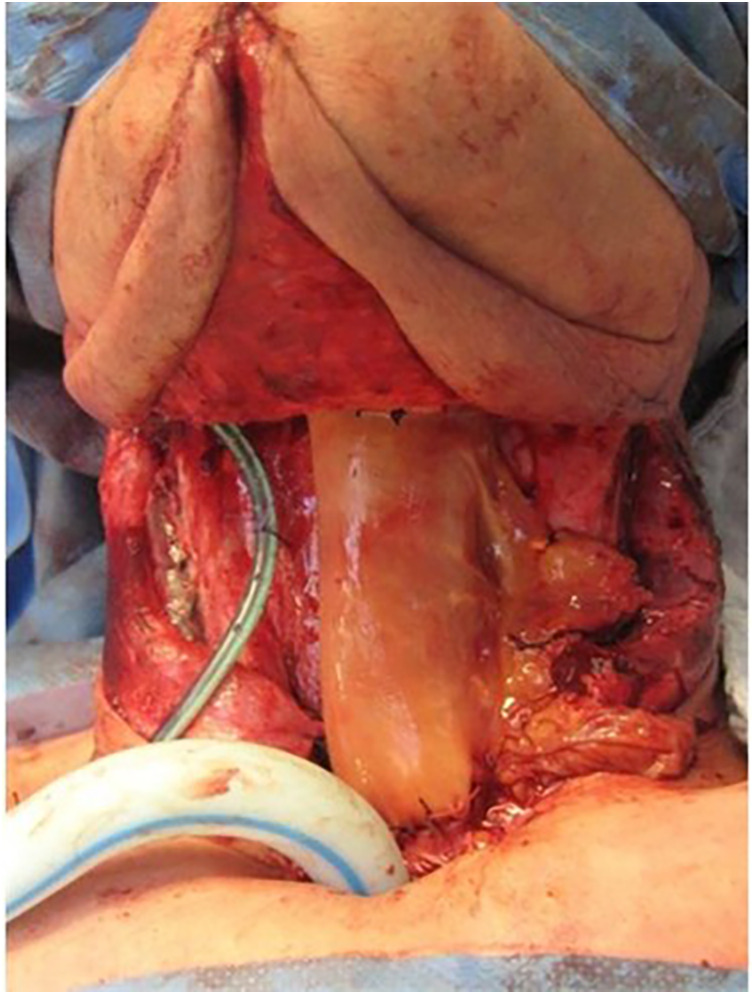
Status after inset of the free jejunal flap. Note the jejunum being stretched between the oropharynx and the esophagus, in order to avoid postoperative kinking.

To ensure safe surgery and successful reconstruction, preoperative hemoglobin levels above 10 g/dl and platelet counts above 50 × 10^3^/µL are considered of paramount importance. The surgical procedure was performed by a multidisciplinary team, including head and neck, reconstructive, and abdominal surgeons. First, a TLP with neck dissection tailored to the nodal status (elective bilateral neck dissection levels II–IV in cN0 cases and ipsilateral modified radical neck dissection in c*N*+ cases, in both primary and salvage setting) was performed by the head and neck surgical team. Moreover, (hemi)thyroidectomy and bilateral central compartment lymph node dissection were standard of care. After completion of the TLP and confirmation of free margins using frozen section analysis, the length of the defect between the oropharynx and the esophagus was measured. Primary insertion of a tracheoesophageal voice prosthesis (TEP) was considered in primary cases if sufficient distance between the caudal resection margin (and thus the distal anastomosis) and the puncture site could be ensured. Subsequently, the abdominal surgical team performed a mini laparotomy and harvested a suitable segment of jejunum (approximately 15 cm), ensuring preservation of its mesenteric arcade and vascular pedicle. Following resection of the jejunal segment, an end-to-end anastomosis was performed and a jejunostomy was created for postoperative feeding. After transposition of the FJT to the neck, flap inset was initiated, taking the direction of jejunal peristalsis into account. To correct for the size mismatch between the oropharynx and the jejunal flap, the cranial end of the FJT was incised opposite the mesentery to maximize preservation of its vascular supply and anastomosed to the pharynx using Vicryl 3-0 or PDS 3-0 (Ethicon, JnJ, Ohio, USA) in two layers (mucosa/muscle layer and serosal layer). After completion of the cranial anastomosis, the flap was stretched towards the esophagus, which was incised vertically along its anterior wall to prevent a circular anastomosis, reducing the risk of stenosis/stricture formation at the level of the distal anastomosis. Redundant jejunal tissue was removed before achieving the distal anastomosis. Typically, a portion of redundant jejunum was preserved, together with its blood supply, and sutured laterally in the neck, where it served as an external flap for postoperative monitoring of flap viability (Figure 1)*.* During jejunal inset, it is of paramount importance to achieve adequate alignment of the mesenterial blood vessels with the recipient blood vessels in the neck to facilitate successful microvascular anastomoses and to maintain sufficient tension on the jejunal segment to avoid postoperative kinking and resulting dysphagia (Figure 2). The distal anastomosis was completed using Vicryl 3-0 or PDS 3-0, again in two layers. As an additional technical measure to prevent postoperative kinking, the jejunal flap was fixed to the prevertebral fascia with two or three sutures. After completion of jejunal flap inset, the reconstructive surgical team performed the microvascular anastomosis.

### Postoperative management and functional rehabilitation

All patients were postoperatively transferred to the post-anesthesia care or intensive care unit for close hemodynamic and flap monitoring for at least 12 h. A strict nil per os policy (NPO) was routinely imposed until contrast swallow fluoroscopy on postoperative day 7 confirmed anastomotic integrity. In patients with previous RT, swallow fluoroscopy was scheduled on postoperative day 10. After exclusion of anastomotic leakage, progressive oral intake of a (semi)liquid diet was initiated, supported and guided by dieticians and speech therapists. Until resumption of oral intake, enteral feeding was provided via a nasogastric, jejunostomy or gastrostomy feeding tube. If primary TEP was not achieved, an electrolarynx or secondary TEP was used for speech rehabilitation.

### Clinical follow-up

Patients were postoperatively monitored at regular intervals. Follow-up appointments were scheduled every two months during the first two years, every three months in the third year, every four months in the fourth year, and every five months in the fifth year. A CT scan of the neck was performed four months after surgery, with additional scans at one and two years postoperatively. To detect distant disease, chest CT or PET-CT was routinely performed annually.

### Oncological and functional outcomes and statistical analysis

Successful complete oral intake was defined as independence from enteral feeding, regardless of food consistency, and successful voice rehabilitation was defined as achievement of the ability to communicate effectively in a one-to-one conversation (“functional” speech) using an electrolarynx or TEP but without the need for additional aids (such as writing). Additionally, the functional oral intake scale (FOIS) was used for follow-up assessment of oral intake (9). Complications were classified as early (in-hospital) or late (out of hospital). All statistical analyses were performed using SAS software (version 9.4 of the SAS System for Windows). Oncological outcomes were assessed using Kaplan–Meier survival estimates, including overall survival (OS), disease-free survival (DFS), disease-specific survival (DSS), and locoregional recurrence-free survival (LRFS). To account for competing risks in disease-specific survival, the cumulative incidence function (CIF) was applied, considering death from other causes as a competing event. Group comparisons were performed using the Mann–Whitney *U*-test for continuous or ordinal variables, or the Fisher exact test for categorical variables. Comparison of survival estimates between primary and salvage groups was performed using log-rank testing. A *p*-value < 0.05 was considered statistically significant for all analyses.

## Results

### Patient characteristics

Twenty-two, predominantly male, patients were included. Mean age at the time of surgery was 65.6 years. All patients reported a history of either past or current tobacco use and alcohol consumption. Main symptoms at presentation were oropharyngeal pain (54.5%) and dysphagia (40.9%), with mean FOIS score of 6.2 (range 3–7, SD 1.07). Comorbidities were quantified and reported using the Charlson Comorbidity Index (CCI), a validated scoring system that predicts 10-year survival based on the presence and severity of comorbid conditions (10). The mean reported CCI score was 4.7 (range 2-10, SD 1.7) which corresponds to an estimated comorbidity-related 10-year survival of approximately 31%, illustrating the substantial comorbidity burden in this patient cohort. Preoperative hemoglobin levels ranged from 10.3 to 16.0 g/dL. Mild anemia was present in 4 patients (18.2%) with hemoglobin levels below 12 g/dL (range 10.3–11.9 g/dL). No patient had severe anemia requiring preoperative blood transfusion or postponement of surgery. Patient characteristics are summarized in Table 1.

**Table 1:** Overview of patients characteristics.

Patient characteristics		n/N (%)
Demographics		
Female		4/22 (18.2)
Male		18/22 (81.8)
Age (years)	Mean	65.6
	SD	9.7
	Range	46.8–77.8
Smoking History		
Active smoker		7/22 (31.8)
Ex-smoker		15/22 (68.2)
Pack years	Mean	30.2
	SD	14.7
	Range	4.0–60.0
Alcohol Use		
Occasionally drinks		4/22 (18.2)
Active heavy drinker		8/22 (36.4)
Past heavy drinker		10/22 (45.5)
ASA Score		
ASA II		5/22 (22.73)
ASA III		14/22 (63.64)
ASA IV		3/22 (13.64)
Charlson comorbidity score		
CCI – Score:	Mean	4.7
	SD	1.7
2		2/22 (9.1)
3		4/22 (18.2)
4		2/22 (9.1)
5		8/22 (36.4)
6		5 (22.7)
10		1 (4.6)
Physical Status		
BMI: <18.5		5/22 (22.7)
BMI: 18.5–25		8/22 (36.4)
BMI: 25–30		7/22 (31.8)
BMI: >30		2/22 (9.09)
Hemoglobin: <12.5 g/dL		7/22 (31.8)
≥12.5 g/dL		15/22 (68.2)
FOIS score pre-operative		
3		1/22 (4.5)
5		4/22 (18.2)
6		5/22 (22.7)
7		12/22 (54.6)
	Mean	6.2
	SD	1.1
	Range	3.0–7.0
Preoperative Status		
Tracheostomy pre-operative		3/22 (13.6)
Tube feeding pre-operative		1/22 (4.6)

Heavy drinkers were defined as consuming more than 2 daily units daily for men and more than 1 unit for women. ASA, American Society of Anesthesiologists; BMI, Body Mass Index and FOIS, Functional Oral Intake Scale.

### Tumor characteristics

Eight patients (36.4%) underwent primary TLP and 14 (63.6%) underwent salvage TLP. Indications for salvage TLP were residual and recurrent hypopharyngeal cancer after primary (C)RT (*n* = 1 and 5 respectively) and hypopharyngeal second primary tumors (*n* = 8). Half of the salvage patients (*n* = 7, 50.0%) had received RT only, while 42.9% (*n* = 6) had undergone CRT. Additionally, one patient (7.1%) had a previous history of supraglottic carcinoma, treated with transoral robotic surgery with bilateral neck dissection and adjuvant RT. In the salvage cohort, the time interval between initiation of (chemo)radiotherapy and salvage surgery ranged from 15 to 296 months (mean 80.5 months, median 70 months).

Extension of the hypopharyngeal tumor into the esophagus and the oropharynx was observed in 3 (13.6%) and 1 (4.5%) patient respectively. Detailed tumor classifications and stage distributions, are presented in Table 2. In the primary group, all patients were clinically staged as stage IVa. The salvage group showed a wider distribution, with tumor classification ranging from clinical stages II to IVb. All patients were free of distant metastases at presentation. Postoperative pathological assessment revealed poorly differentiated SCC in 50.0% of patients, moderately differentiated SCC in 40.9% and highly differentiated SCC in 1 patient (4.5%). Lymphovascular and perineural invasion were both observed in 14 of 22 patients (63.6%). Surgical margins were considered free (>5 mm) in 2 (9.1%) patients, close (≤5 mm) in 16 (76.2%) and positive (presence of tumor cells at the resection margin) in 4 (19.1%) patients.

**Table 2:** Overview of tumor characteristics.

Tumor characteristic	Primary Group *n* (%)	Salvage Group *n* (%)
Clinical tumor classification		
cT2	0	2 (14.3)
cT3	2 (25.0)	6 (42.9)
cT4a	6 (75.0)	5 (35.7)
cT4b	0	1 (7.1)
Clinical nodal classification		
cN0	2 (25.0)	10 (71.4)
cN1	0	2 (14.3)
cN2b	1 (12.5)	0
cN2c	5 (62.5)	2 (14.3)
Clinical tumor stage		
II	0	1 (7.1)
III	0	5 (35.7)
IVa	8 (100.0)	7 (50.0)
IVb	0	1 (7.1)
Pathological tumor classification		
pT2	0	4 (28.6)
pT3	0	2 (14.3)
pT4a	8 (100.0)	8 (57.1)
Pathological nodal classification		
pN0	0	9 (64.3)
pN1	0	1 (7.1)
pN2a	0	1 (7.1)
pN2c	1 (12.5)	0
pN3b	7 (87.5)	2 (14.3)
Nx	0	1 (7.1)
Pathological tumor stage		
II	0	3 (21.4)
III	0	1 (7.1)
IVa	1 (12.5)	7 (50.0)
IVb	7 (87.5)	2 (14.3)
IVc	0	0
Undefined	0	1 (7.1)

Among the 12 patients initially staged as cN0 preoperatively, 8 remained pN0 postoperatively, while 4 were upstaged to pN+ upon histopathological analysis. In total, 12 (54.5%) patients had pathology-confirmed lymph node metastases in the neck (pN+); the mean number of positive lymph nodes was 8 (range 1–40) and extracapsular extension was observed in the majority of pN+ patients (10 of 12 patients, 83.3%). In pN+ patients, 75.0% had cervical metastases in both ipsilateral and contralateral lateral neck compartments. Isolated ipsilateral lateral compartment involvement (levels II-IV) was observed in 16.7% of cases, while isolated central compartment metastases (levels VI-VII) occurred in 8.3%.

### Surgery/treatment characteristics

In cN+ cases (*n* = 10, 45.5%), the involved neck was treated with modified radical neck dissection, including resection of the sternocleidomastoid muscle (*n* = 10), the internal jugular vein (IJV) (*n* = 4, 18.2%), the accessory nerve (*n* = 4, 18.2%) and the common carotid artery (*n* = 1, 4.5%) according to the extent of nodal disease. In the cN0 neck, bilateral elective neck dissection of levels II-III-IV were routinely performed. Moreover, all patients underwent a bilateral central compartment neck dissection.

Microvascular arterial and venous anastomoses for the jejunal interposition graft were established using different configurations. The arterial anastomosis was most frequently performed end-to-end (81.8%, *n* = 18) with the superior thyroid artery as the most frequently used recipient vessel (54.5%, *n* = 12), followed by the inferior thyroid artery (18.2%, *n* = 4). Other recipient arteries used were the lingual and facial arteries (end-to-end anastomosis) and the internal and external carotid arteries (end-to-side anastomosis), each in one patient (4.5%). In two cases (9.1%), the recipient artery was not specified in the operating report. For the venous anastomosis, an end-to-end connection with a branch of the IJV was performed in 63.6% (*n* = 14) of cases, while an end-to-side anastomosis on the IJV was performed in 22.7% (*n* = 5).

Postoperatively, all primary patients received adjuvant RT (*n* = 2) or concurrent CRT (*n* = 6). Moreover, 1 salvage patient underwent postoperative re-irradiation and 1 was treated with adjuvant immunotherapy. Overall, 54.5% of patients did not receive any form of adjuvant therapy.

### Hospitalization and complications

Mean hospital stay was 37 days (media*n* = 28 days, range 12-117 days, SD = 25 days). No in-hospital mortality occurred. The most frequent complication was fistula formation: pharyngocutaneous fistulas (PCF) developed in 6 patients (27.3%) and were more frequently observed in salvage cases (*n* = 5 or 35.7%) compared to primary cases (*n* = 1 or 12.5%). PCFs were treated conservatively (*n* = 2) or with surgical reintervention using a pectoralis major flap (PMF) to cover the fistula (*n* = 4). All patients requiring surgical reintervention were salvage cases. Patients who developed a PCF had a significantly longer mean hospital stay compared to patients without fistula formation (50.8 days vs. 18.2 days; *p* < 0.001). Other in-hospital head and neck surgical complications were FJT failure due to thrombosis of the venous anastomosis (*n* = 1, requiring salvage reconstruction with gastric pull-up), post-operative hemorrhage (*n* = 6, 4 managed conservatively, 2 salvage cases requiring surgical reintervention), wound infection (*n* = 6, 5 treated with intravenous antibiotics, 1 salvage case requiring surgery), neck wound dehiscence (*n* = 2) and blow-out of the common carotid artery secondary to a PCF in a salvage patient (*n* = 1, successfully controlled with endovascular embolization without resulting neurological deficits). In-hospital complications related to the jejunal harvest were wound dehiscence of the mini-laparotomy wound (*n* = 1), intra-abdominal hemorrhage (*n* = 1) and prolonged ileus (*n* = 1). Out-of-hospital complications included temporary (≤6 months) and permanent (>6 months) hypoparathyroidism in 4 (18.2%) and 8 (36.4%) patients, respectively. Accessory nerve paralysis/paresis was reported in 2 patients with an intra-operatively preserved nerve. Intestinal obstruction developed in 2 patients. Additionally, rare complications included deep vein thrombosis, pulmonary embolism, aspiration pneumonia and severe electrolyte disturbances (e.g., hypocalcemia, hyponatremia), each occurring once. Six patients were readmitted due to late complications, with a mean readmission duration of 7 days. Postoperative head and neck-related complications for both primary and salvage groups are reported in Table 3. Surgical revision, either for management of PCF, postoperative hemorrhage, postoperative infection/abcedation or FJT failure proved more common in salvage cases (8/14 or 57.1%) than in primary cases (*n* = 0).

**Table 3:** Overview of postoperative complications in primary and salvage groups.

Complications	Primary group (*n* = 8)	Salvage group (*n* = 14)
PCF		
Conservative treatment	1	1
Surgical reintervention	0	4
FJT failure	0	1
Postoperative hemorrhage		
Conservative treatment	3	1
Surgical reintervention	0	2
Wound infection, conservative		
Conservative treatment	3	2
Surgical reintervention	0	1
Carotid blowout	0	1
Hypoparathyroidy		
Temporary	1	3
Permanent	4	4
Wound dehiscence	1	1
Accessory nerve paralysis	2	0

FJT, free jejunal transfer; PCF, pharyngocutaneous fistula.

### Oncological outcomes

The mean duration of follow-up for the entire patient population was 3.9 years (median 4.8 years, range 0.4–5.4 years, SD = 1.6 years). Death occurred during follow-up in 14 patients (63.6%) and was disease-related in 10 patients (45.5%). Thirteen patients (59.1%) developed disease recurrence: 6 patients (27.3%) had locoregional recurrence, 6 patients (27.3%) presented with distant disease and 1 patient (4.5%) had both distant and locoregional disease. The mean time to recurrence was 9 months. Treatment strategies for disease recurrence were chemotherapy alone (*n* = 2), immunotherapy alone (*n* = 1) and a combination of chemo-and immunotherapy (*n* = 6).

**Figure 3:**
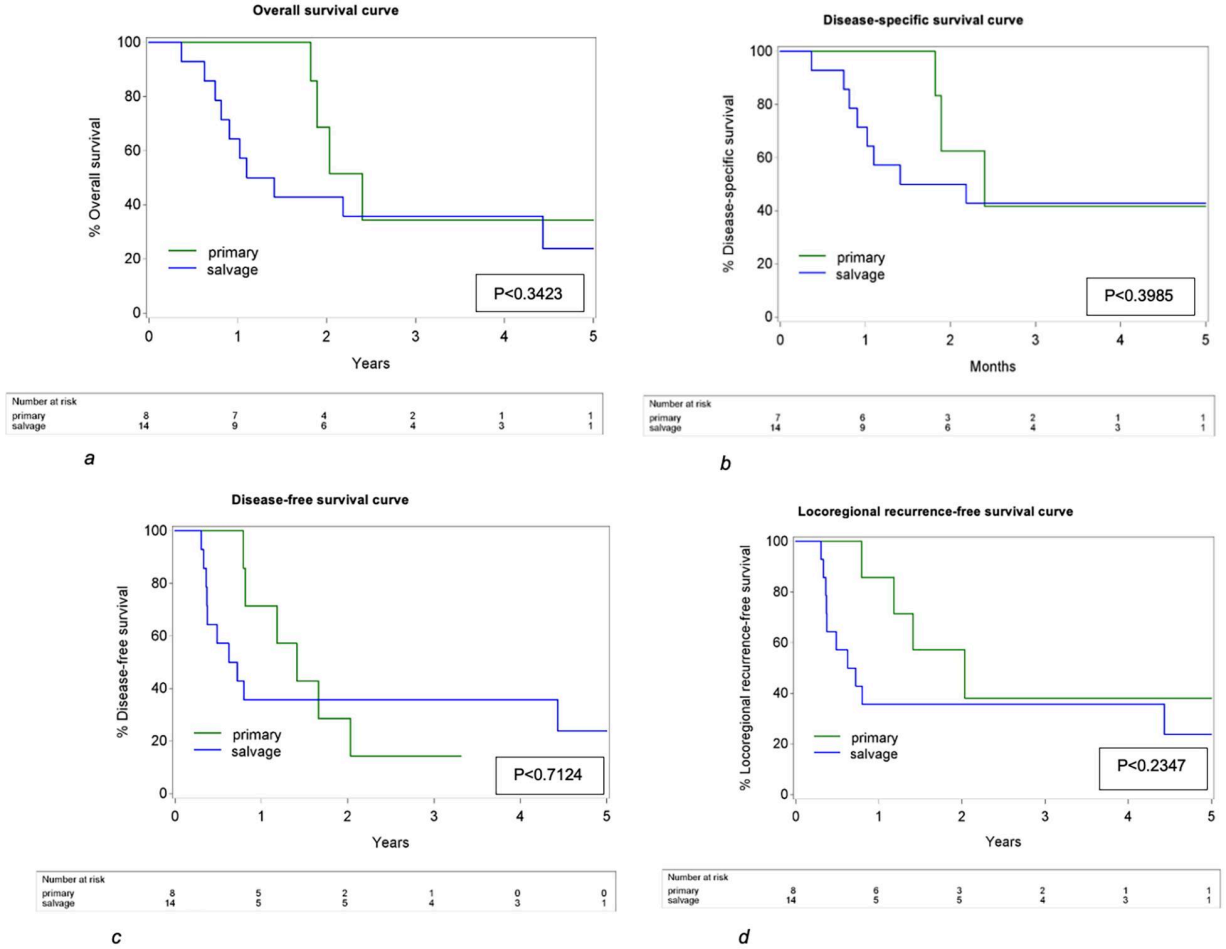
**(a)** kaplan–meier curve for overall survival outcome, with comparison between primary and salvage group. **(b)** Kaplan–Meier curve for Disease-specific survival outcome, with comparison between primary and salvage group. **(c)** *Kaplan–Meier curve for Disease-free survival outcome, with comparison between primary and salvage group*. **(d)** Kaplan–Meier curve for Locoregional recurrence-free survival outcome, with comparison between primary and salvage group.

Kaplan–Meier survival analysis showed 2- and 5-year OS rates of 52.3% and 26.9% respectively for the entire population. In the primary group, 2-and 5-year OS rates were 68.6% and 34.3% and 42.9% and 23.8% in the salvage group, respectively, with no significant difference (*p* = 0.3423). (Figure 3a) Two-and five-year DSS rates were 62.5% and 41.7% in the primary group vs. 50% and 42.9% in the salvage group, respectively. As for OS, a trend towards better DSS in the primary group compared to the salvage group was observed (*p* = 0.3985). (Figure 3b) Two- and five-year DFS rates were 35.7% and 23.8% in the salvage group, while a 2-year DFS of 28.6% was observed in the primary group (*p* = 0.7). (Figure 3c). Finally, 2-and 5-year LRFS rates were 57.1% and 38.1% in the primary groups vs. 35.7% and 23.8% in the salvage group, respectively (*p* = 0.299) (Figure 3d).

### Functional outcomes

**Figure 4:**
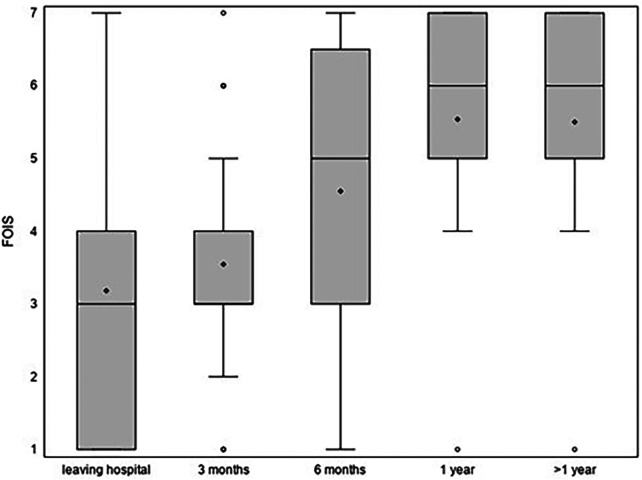
Box plots with FOIS scores at five time points: at leaving hospital, 3 months, 6 months, 1 year, and more than 1 year postoperatively. Median FOIS scores increase over time, indicating improved outcomes.

In the immediate postoperative phase, patients were dependent for their caloric intake on jejunostomy feeding (*n* = 10, 45.5%), gastrostomy feeding (*n* = 7, 31.8%) or total parenteral nutrition (*n* = 5, 22.7%). The mean duration of NPO was 16.3 days (range: 8–34 days). Upon discharge, 40.9% of patients (*n* = 9) had achieved complete peroral caloric intake. Incomplete peroral caloric intake with additional tube feeding was necessary in 7 patients (31.8%). Six patients (27.7%) had no oral intake at discharge and remained fully dependent on tube feeding. Initiation of oral intake differed significantly between groups: all primary patients resumed oral intake during hospitalization, with or without additional tube feeding, compared with 50% of salvage patients (*p* = 0.02). During follow-up, oral intake improved among the surviving patients. At three months postoperatively, 45.5% (*n* = 10) had achieved complete peroral caloric intake, 9 (40.9%) required a combination of oral and tube feeding and 3 patients (13.6%) remained fully tube dependent. After six months, 14 out of 20 surviving patients (70%) achieved full peroral caloric intake with an increasing portion reaching a diet without any restrictions (FOIS 7): 7 patients improved from an initial FOIS score of 3/4 one month postoperatively to a score of 6/7 six months postoperatively. Figure 4 illustrates the improvement of FOIS score evolution over time among surviving patients.

However, 3 patients (15%), all treated in the salvage setting, required continued combined oral-tube feeding: 1 patient developed a dilation-resistant, high grade stenosis of the pharyngogastric anastomosis after gastric transposition as a salvage treatment for FJT failure. In the 2 other patients, dysphagia was considered functional (unfavorable peristalsis of the FJT), as no abnormal anatomical configuration or stenosis of the FJT were observed on swallow fluoroscopy. Additionally, another 3 salvage patients (15%) remained fully dependent on tube feeding: 2 due to persistent postoperative PCF's, preventing resumption of oral intake, and 1 due to total dysphagia, likely secondary to functional impairment and unfavorable peristalsis, as no organic stenosis was observed on swallow fluoroscopy and pharynghoesophagoscopy. This case required multiple interventions, including endoscopic dilation, vertical midline incision above the tracheostomy site for decompression of the jejunal interposition, and reconstruction with a myocutaneous PMF. Despite these measures, the patient remained permanently dependent on enteral tube feeding.

At last follow-up, complete peroral caloric intake, independent of enteral support, was achieved in 15 patients (68.2% of the total population). During follow-up, 18 of 22 patients (81.8%) did not require any additional surgical intervention for dysphagia. Endoscopic balloon dilation was performed in 2 patients (9.1%), and 1 patient (4.5%) underwent secondary augmentation of the jejunal lumen using a RFFF. Of interest, minor kinking of the jejunal segment, diagnosed on swallow fluoroscopy, was observed in only 1 patient with moderate postoperative dysphagia (FOIS score of 5, 6 months postoperatively).

Concerning speech rehabilitation, 52.3% of patients successfully regained functional speech. Among these patients, speech production with a TEP was achieved in 63.6% of cases and with an electolarynx in 36.4%. A TEP was placed in 12 patients (54.6%), either as a primary or secondary procedure. Among primary TEP patients, 5 (83.3%) achieved functional speech, while 1 patient had an unknown outcome. Among secondary TEP patients, only 2 out of 6 (33.3%) achieved functional speech. The main cause of failure was excessive secretion pooling in the jejunum interposition, resulting in a “wet” voice, limiting speech intelligibility. A group of patients used electrolarynx (*n* = 5, 22.7%) as an alternative method of rehabilitation. Among these, 4 patients (80%) achieved functional speech, while 1 patient (20%) could not produce intelligible speech due to radiotherapy-induced fibrosis of the neck.

## Discussion

The purpose of this retrospective study was to evaluate oncological and functional outcomes after TLP with FJT reconstruction. In HSCC, early and intermediate stages are generally managed with organ-preserving CRT, whereas advanced tumors often require TLP due to poor functional preservation and insufficient disease control (11, 12). Several studies have confirmed that TLP remains the gold standard for locally advanced or residual HSCC, or for locoregional recurrence after RT/CRT (4, 5, 13).

Our findings demonstrate that FJT is a viable reconstructive option after TLP, restoring the anatomical continuity of the digestive tract. The success rate of FJT reconstruction is high (95.5%), with only one patient (4.5%) requiring secondary gastric pull-up reconstruction due to flap failure. This is consistent with previous studies, which report flap failure rates ranging from 0% to 17.4% (14–18). Moreover, FJT reconstruction proved to be safe, with no in-hospital mortalities compared to reported perioperative mortality rates of 3%–9% (16–22). This is also lower than the 10% mortality previously reported for gastric transposition in our own series (6). Effective perioperative management and a multidisciplinary approach are warranted to achieve optimal results, as complications after TLP with FJT are frequent and have important clinical consequences. PCF formation occurred in 27.3% of our patients, which falls within the range reported in the recent systematic review by Mortaja et al., who analyzed 3.191 FJT reconstructions with PCF rates ranging from 0% to 35.29%. (23) Importantly, this wide range reflects the heterogeneity of the included patient populations. Mortaja et al. reported a relative risk of 2.46 for PCF formation in salvage cases, specifically in patients who had previously undergone RT. As our patient group consisted of a substantial proportion of salvage cases (63.6%), this may explain the relatively high overall PCF rate. PCF formation was more commonly observed in salvage cases compared to primary cases (35.7% vs. 12.5%) and frequently necessitated surgical revision in the salvage group. When compared with similar cohorts including salvage cases, our PCF rates are comparable to those reported by Keereweer et al. and by Park et al. (35.3% and 29.5% respectively) (24, 25). Moreover, FJT reconstruction performs favorably when compared to other reconstructive techniques: Tan et al. reported fistula rates of 50% after TLP with reconstruction using radial forearm free flaps (RFFF) and 33% when using anterolateral thigh (ALT) flaps, compared to 30% for FJT reconstruction. (26) Similarly, in the study by Keereweer et al. gastric pull-up reconstruction was associated with PCF rate of 52.6%, compared to 35.3% after FJT reconstruction. (24) However, in our experience, TLP with gastric transposition did not show a higher incidence of postoperative PCF (8.3% vs. 27.3% in FJT patients) (6). Additionally, we observed a significant increase in length of hospitalization among patients who developed a PCF, with an average stay of 50.8 days compared to 18.2 days in patients without PCF formation (*p* < 0.001). This illustrates the clinical importance of PCF prevention and early management, particularly in the salvage setting. Of interest, the rate of head/neck surgical revision, either for management of PCF, postoperative hemorrhage, postoperative infection/abcedation or FJT failure was as high as 36.4% and was concentrated in the salvage patient group (57.1% of salvage cases vs. 0% of primary cases). This finding is consistent with the well-known negative effect of prior radiotherapy on postoperative healing and illustrates the challenges of salvage surgery for hypopharyngeal cancer. Although FJT reconstruction may yield less PCF's and less strictures compared to reconstruction with fasciocutaneous free flaps, the requirement for a laparotomy and its abdominal complications (e.g., ileus, wound dehiscence, and late intestinal obstruction) are not uncommon and are considered significant drawbacks (26). As abdominal complications may be exacerbated by previous intra-abdominal surgery or the presence of liver or intestinal diseases, these constitute a (relative) contraindication for jejunal flap harvesting (27). Therefore, pre-operative multidisciplinary discussion and postoperative multidisciplinary follow-up, including the abdominal surgery team, remain important for early diagnosis and management of these complications, which are less familiar to the head and neck surgeon.

Concerning the oncological outcomes, estimated survival rates remain poor in patients with HSCC, with 2-year OS of 52.3%, decreasing to 26.9% at 5 years. These findings are consistent with previously reported outcomes of 2-year OS ranging from 39.5% to 83% and 5-year OS rates from 20% to 54% (6, 18–21). In our patient group, no significant differences in oncological outcomes were observed between primary and salvage groups. These poor outcomes reflect the substantial comorbidity burden of the HSCC patient population, as reflected in our series by a high CCI, in addition to the known aggressivity of the disease.

Regarding functional outcomes, 40.9% of patients achieved complete peroral caloric intake without additional tube feeding upon discharge, which increased during follow-up to 68.2%. Moreover, the consistency of the peroral diet progressed towards a (near) normal diet (FOIS scores 6 and 7) in an substantial portion of the population, as illustrated by the gradually increasing FOIS scores. These outcomes align with swallowing success rates reported in the literature: 61.9% to 98%, although these figures should be interpreted with caution as swallowing success is often defined as achievement of peroral feeding, without considering diet consistency (15–17, 28). Furthermore, Chan et al. reported return to a normal diet in 61.9% of patients following FJT reconstruction, compared to 38.1% in patients undergoing ALT flap reconstruction and 35.8% in those with PMF reconstruction, further highlighting the advantage of the jejunal flap in swallowing rehabilitation. The physiological properties of FJT, such as its mucosal surface and peristaltic capability, contribute to these outcomes. (23) Although considered an advantage of the FJT over fasciocutaneous free flaps, autonomous peristalsis may also negatively affect postoperative functionality, with patients reporting dysphagia and sudden blockage of ingested foods and liquids. In some cases, this may impair oral feeding to such an extent that (additional) tube feeding remains necessary. Although (serial) dilatations may be useful for treating anastomotic stenoses, functional, peristalsis-related dysphagia typically does not respond well to dilation. Perioperative injections of botulinum toxin in the muscular layer of the jejunal segment may safely and effectively alleviate postoperative swallowing dysfunction (29). Another potential cause of postoperative dysphagia is gravitational kinking of the jejunal segment, resulting in a siphon-like configuration. As secondary correction by shortening the jejunal interposition is technically demanding, prevention of postoperative kinking, by ensuring sufficient tension on the jejunum during inset and fixation to the prevertebral fascia, is essential. Additionally, a frequent cause of dysphagia in TLP is neopharyngeal stenosis; with reported rates between 10%–31%. (30–33) In our population, no stenoses were observed after successful FJT. However, one patient with failed FJT who was salvaged with gastric transposition, developed a high grade, dilation resistant pharyngogastric stenosis, impairing oral intake. Of interest, all patients with severe postoperative dysphagia requiring tube feeding were treated in the salvage setting. This underscores the negative effect of prior head and neck irradiation on functional outcomes after FJT, likely due to radiation induced fibrosis and pre-existing swallowing dysfunction, as well as impaired healing capacity leading to higher complication rates such as PCF formation. In cases of persisting postoperative dysphagia, swallow fluoroscopy should be performed to differentiate between functional and anatomical causes. If no underlying anatomical cause is identified, additional high-resolution pharyngo-esophageal manometry may be considered. Postoperatively, speech and language pathologists initiated speech rehabilitation by either TEP or electrolarynx, depending on the patient's individual situation. Ultimately, 52.3% regained functional speech; which is comparable to reported rates ranging from 22% to72% (34, 35). It is important to note that our cohort included a high proportion of salvage patients, in whom speech rehabilitation is generally more complex: post-RT tissue fibrosis or previous surgeries can limit the possibility of placing a TEP or can hamper the use of an electrolarynx. (23) In our cohort, primary TEP was more likely to result in successful speech rehabilitation than secondary TEP placement (83.3% vs. 22%). This difference is most likely due to selection bias, as salvage patients and patients with less favorable tissue characteristics, which independently negatively affects speech rehabilitation success, were more likely to undergo secondary TEP placement. The main reason for TEP failure was excessive secretion production by the jejunal flap and secretion stasis, which impaired effective phonation. An electrolarynx was used as an alternative for TEP in 22.7% of cases, and proved effective in 80%. These findings are consistent with the literature, with Keereweer et al. (24) reporting functional speech rates of 52% with electrolarynx and 43% with TEP, and Sharp et al. (30) reporting success rates up to 80% with electrolarynx. However, success with the electrolarynx is highly patient-dependent and can be limited by skin fibrosis following RT.

Overall, these findings suggest that reconstruction with FJT achieves acceptable functional outcomes. However, when compared qualitatively and quantitatively with outcomes after total laryngectomy without circumferential pharyngectomy, result remain inferior, highlighting the limitations of FJT. In the series by Harris et al., approximately 80% of patients undergoing primary total laryngectomy achieved full oral intake with a FOIS score > 4, compared to 68.2% in our cohort (36). Regarding speech rehabilitation, Murariu et al. reported that after primary total laryngectomy, 68% achieved successful TEP speech at 12 months, which is superior to success rates achieved after TLP with FJT (37).

In our previous TLP with gastric transposition series, 82.7% of patients achieved complete oral intake, compared to 68.2% after TLP with FJT in the current cohort, with higher tube dependence in salvage cases for both techniques. Regarding speech rehabilitation, the gastric pull-up group achieved an overall success rate of 66%, with 51.5% using a voice prosthesis and 48.5% using an electrolarynx, whereas in the TLP + FJT group, 50% of patients regained functional speech, of whom 63.6% achieved speech via TEP and 36% via electrolarynx. However, as only 86% of patients were evaluable for functional outcomes in the gastric pull-up series, these results may be slightly overestimated (6).

We recognize several limitations. The first is the retrospective design of this study, introducing an inevitable risk of selection bias. Although consecutive patients were included, patient inclusion was dependent on the availability of medical records. As a consequence, the included patients may not fully represent the entire patient population. Moreover, given the long inclusion period, evolution in management of patients undergoing TLP with FJT is evident. Although the surgical protocol did not change throughout the observed period, perioperative and postoperative supportive care evolved, which may have contributed to reduced complication rates and improved survival in more recently treated patients. Additionally, the shift from 3D-conformal radiotherapy to intensity-modulated radiotherapy may have influenced postoperative complication rates (e.g., PCF) and functional outcomes, in both salvage cases and primary cases treated with surgery and adjuvant therapy. Second, our sample size was limited, which reduces the statistical power of our findings and limit generalizability. Moreover, the salvage subgroup is heterogeneous, including patients with residual, recurrent and second primary tumors which are to be considered having distinct postoperative complication profiles and survival expectations, e.g., residual tumor is considered a significant negative prognostic factor after salvage surgery for various head and neck cancer sites (38). However, given the small salvage population, no comparisons in complication profiles and oncological outcomes between salvage subgroups could be made. Another limitation was the lack of objective assessment data on speech quality and swallowing function (e.g., videofluoroscopic swallowing studies and manometry). While validated scales such as the FOIS were used to monitor oral intake, standardized and quantitative assessment methods for speech outcomes were not consistently available or documented, making comparisons with other studies difficult. Due to the small number of patients, multivariate analysis to identify independent predictors for outcome was not feasible. Therefore, any observed trends should be interpreted with caution. There is a clear need for future prospective research with larger cohorts and standardized assessment tools to validate these findings.

## Conclusion

Free jejunal flap reconstruction proves a highly successful technique for restoring anatomical continuity of the upper digestive tract following TLP. However, long-term survival remains poor, largely due to the aggressive nature of HSCC and the substantial comorbidity burden of this patient population. Functional outcomes show acceptable swallowing rehabilitation. However, speech restoration remains a significant challenge. To optimize the functional outcomes, careful patient selection and structured postoperative multidisciplinary rehabilitation, including dietary specialists and speech and language pathologists, are essential.

## Data Availability

This work was presented as an oral communication during the Belgian ENT society conference (Brussels, 15th February 2025).

## Data availability statement

The raw data supporting the conclusions of this article will be made available by the authors, without undue reservation.
